# Characteristics, treatments, and outcomes of patients with necrotizing soft tissue infections: a Dutch multicenter cohort study

**DOI:** 10.1007/s00068-025-03000-8

**Published:** 2025-10-28

**Authors:** Jesse de Haan, Lidewij M.F.H. Neeter, Jaco Suijker, Paul P.M. van Zuijlen, Anouk Pijpe, Annebeth Meij-de Vries, NSTI Knowledge Collaborative Group, Jaap Bonjer, Marianne K. Nieuwenhuis, Cornelis H. van der Vlies, Esther M.M. van Lieshout, Kornelis J. Ponsen, Maartje Terra, Pieta Krijnen, Nico L. Sosef, Jasper Winkelhagen, Steve M.M. de Castro, Bas A. Twigt, Lisca Wurfbain

**Affiliations:** 1 Alliance of Dutch Burn Care (ADBC), Burn Centre, Red Cross Hospital, Beverwijk, 1942 LE The Netherlands; 2 Department of Plastic, Reconstructive & Hand Surgery, Amsterdam UMC Location Vrije Universiteit, Amsterdam, 1081 HV The Netherlands; 3 Amsterdam Movement Sciences (AMS), Tissue Function and Regeneration, Amsterdam, 1081 HV The Netherlands; 4 Department of Plastic, Reconstructive & Hand Surgery, Red Cross Hospital, Beverwijk, 1942 LE The Netherlands; 5 Paediatric Surgical Centre, Emma Children’s Hospital, Amsterdam UMC Location University of Amsterdam, Amsterdam, 1105 AZ The Netherlands; 6 Department of Surgery, Red Cross Hospital, Beverwijk, 1942 LE The Netherlands

**Keywords:** Necrotizing soft tissue infection, Necrotizing fasciitis, Group A Streptococcus, Mortality, Amputation

## Abstract

**Purpose:**

Necrotizing Soft Tissue Infections (NSTI) are associated with high mortality and morbidity. This study aimed to gain insights into the patient-, disease-, and treatment characteristics, as well as the clinical outcomes of NSTI patients in the Netherlands, contributing to the global knowledge of this disease.

**Methods:**

This study analyzed the NSTI Knowledge Project cohort, comprising 271 patients who were treated for acute NSTI in 11 hospitals across the Netherlands between 2013 and 2017.

**Results:**

Most patients (61%) presented with early-stage NSTI symptoms, such as pain or erythema. Intensive care unit admission was required in 83%, with a median stay of 5 days (interquartile range 2–11). The median time from hospital admission to debridement was 8 h (interquartile range 4–23). Group A Streptococcus was cultured in 41% of patients. Extremity amputation was required in 12%, and the in-hospital mortality rate was 21%. Patients presenting with early-stage symptoms who were misdiagnosed (60%) had a significantly higher in-hospital mortality rate (30%) than those correctly diagnosed (10%; p = *.003*).

**Conclusion:**

In the Netherlands, NSTI patient and disease characteristics vary considerably. With one in five patients dying and one in eight patients undergoing a major amputation, interventions leading to rapid diagnosis and treatment are urgently needed.

## Introduction

Necrotizing Soft Tissue Infections (NSTI) are rapidly progressive infections associated with substantial mortality and morbidity [1–3]. NSTI encompasses a spectrum of necrotizing soft tissue infections, including necrotizing cellulitis, necrotizing fasciitis, and necrotizing myositis. The origin of NSTI can be either monomicrobial, most commonly caused by the Group A Streptococcus (GAS), or from multiple pathogens in polymicrobial infections. Clinically, patients usually present with early localized symptoms such as pain, erythema, and swelling, which may be accompanied by systemic signs such as fever. When the disease progresses, late-stage localized symptoms, such as hemorrhagic bullae and skin necrosis, become apparent. Most patients will develop septic shock, necessitating admission to the intensive care unit (ICU) [4]. Treatment requires surgical debridement, broad antibiotic therapy, and supportive care [5]. The mortality rate of NSTI remains high, varying widely between 7% and 76% [6–8].

National-level knowledge on NSTI is essential for identifying evolving patterns, enabling cross-national comparisons, and ultimately improving patient care and outcomes. Additionally, such research contributes to the global body of knowledge on NSTI. As of now, various nationwide studies on NSTI have been performed [1, 9, 10]. Internationally, a key contribution to this field comes from the INFECT trial (2013–2017), an international prospective study that examined the clinical characteristics of patients with NSTI across various Scandinavian countries [6]. However, the results of this study, along with other national studies, may not be directly applicable to individual countries due to population differences and variations in treatment approaches.

In the Netherlands, several studies have provided valuable insights into the characteristics and outcomes of NSTI. An epidemiological study that combined data from various Dutch databases and individual studies reported that GAS was responsible for approximately 34% to 42% of NSTI cases. This study also found an average annual mortality rate ranging from 23% to 29%, with amputations required in 11% to 14% of patients [11]. Another cohort, including 123 patients treated in the ICUs of five Dutch hospitals, contributed valuable insights by analyzing a wide range of patient and disease characteristics. This study reported a mortality rate of 32% [12]. These studies highlight that patient outcomes are often suboptimal and that there is significant room for improvement.

Therefore, this study aimed to provide a broad perspective on NSTI care in the Netherlands between 2013 and 2017, covering patient characteristics, clinical presentation, microbiological data, treatment approaches, and clinical outcomes, as this might identify areas where outcomes can be improved.

## Methods

### Study design

In this multicenter retrospective study, the complete NSTI Knowledge Project cohort was analyzed. This cohort includes 271 patients who were treated across 11 hospitals (the study centers) in the Netherlands (four academic hospitals; seven peripheral hospitals, of which three with a burn center). This includes patients who were directly admitted to one of the study centers, as well as patients who were initially treated in a non-study center and later referred to a study center. The medical research ethics committee of Amsterdam University Medical Centre determined that our study was not subject to the Medical Research Involving Human Subjects Act (WMO). In all study centers, approval was obtained from the Institutional Review Board or medical research ethics committee. This study followed the Strengthening the Reporting of Observational Studies in Epidemiology (STROBE) criteria for cohort studies [13].

### Study population and data collection

All patients admitted for acute NSTI between January 1, 2013, and December 31, 2017, were included. The diagnosis of NSTI was made based on perioperative assessment by the surgeon or, in cases of uncertainty, on a frozen section biopsy. In operative reports, surgeons typically described macroscopic signs of tissue breakdown beneath the skin, such as necrotic subcutis, fascia, and/or muscle, ‘dishwater’ exudate, a positive finger test, and/or absence of fascial bleeding upon incision.

Patients were retrospectively identified through several methods: (1) a review of electronic patient records using ICD-10 diagnosis codes from the Dutch ICD-10 codebook for necrotizing fasciitis (M72.6), inflammatory diseases of the scrotum (N49.2), and gas gangrene (A48.0), (2) the Dutch Hospital Data registration, (3) the Dutch Burn Centers Repository R3, and (4) search of free text terms in the electronic health record (EHR). All available data on patient-, disease-, and treatment characteristics, as well as clinical outcomes, were extracted from the EHR by three researchers and documented in an electronic case report form using Castor EDC (Castor, Amsterdam, the Netherlands). If a patient was initially admitted to another hospital before being referred to a study center, all available data from the non-study center were also collected. These methods are consistent with those described by Suijker et al. [14].

### Definitions

*Fournier’s gangrene* was defined as a NSTI of the anogenital area [15]. *Early-stage NSTI symptoms* included erythema, localized pain, swelling, and fever. *Late-stage NSTI symptoms* included bullae, blue-grey discoloration, crepitus on palpation, ecchymosis, and skin necrosis. The *portal of entry* was defined as the site or pathway through which a pathogen enters the body and initiates an infection. This can be any break in the skin, such as a cut, surgical wound, or insect bite. NSTI was classified as *monomicrobial* or *polymicrobial* based on the first intraoperative deep tissue cultures, since antibiotic regimens reduce the reliability of subsequent cultures. *Septic shock* was defined based on the SEPSIS-3 criteria, or if a patient passed away upon presentation before adequate resuscitation could be performed [16]. *Debridement* was defined as the surgical removal of all non-viable tissue down to clearly bleeding, viable layers. A *skin-sparing surgical approach* was documented if it was explicitly stated in the operative note of the first debridement surgery, or if the procedure was clearly described as preserving the skin over the affected deep tissue layers. All other procedures were documented as *non-skin-sparing*.

### Data analyses

Descriptive statistics were used to describe patient characteristics, disease characteristics, treatments, and clinical outcomes. Normality was assessed graphically and using the Shapiro-Wilk test. Continuous variables were reported as median and interquartile range (IQR), as most continuous variables did not follow a normal distribution. However, the variable age followed a normal distribution and was therefore presented as mean and standard deviation (SD). Categorical variables were reported as numbers and percentages. A chi-square test was used to assess statistical differences between categorical variables, while the Mann-Whitney U test was applied for continuous variables. Statistical significance was defined as *p* <.05. Pairwise deletion was used for missing data. All analyses were performed using SPSS (version 28.0, IBM Corp., Armonk, NY, USA).

## Results

### Patient and disease characteristics

A total of 271 patients were included, comprising 267 adults and 4 children (2, 4, 16, and 17 years). A total of 216 (79.7%) patients were admitted directly to a study center, whereas 55 (20.3%) patients were initially admitted to a non-study center and subsequently referred to a study center. In operative reports, necrosis of subcutaneous tissue was described in 63.9%, necrosis of fascia in 73.3%, and necrosis of muscle in 10.0%. Dishwater exudate was described in 21.1%, a positive finger test in 7.6%, and absence of fascia bleeding upon incision in 4.4%. A frozen section biopsy was performed in 6.9% to confirm the diagnosis. The mean age was 56.6 years (SD 15.8), 66.4% were male, and the median Body Mass Index was 25.8 (IQR 23.2–29.3) (Table 1). Two-thirds (67.9%) of patients had comorbidity, of which cardiovascular disease (39.1%) and diabetes (24.4%) were the most common. Patients with diabetes, chronic kidney disease, active malignancy, and immunodeficiency had a significantly higher in-hospital mortality rate than patients without these comorbidities (Table 2). NSTI initially affected the anogenital/gluteal region in 84 patients (31.9%), which included a Fournier’s gangrene in 73 patients. The lower extremity was affected in 79 patients (30.0%), the upper extremity/thoracic region in 52 patients (19.8%), the abdominal region in 31 patients (11.8%), and the head/neck region in 17 patients (6.5%).

**Table 1:** Patient and disease characteristics

	*N* = 271
Demographic data	
Age (years), mean (SD)	56.6 (15.8) *(m = 1)*
Male, *n* (%)	180 (66.4%)
BMI (kg/m^2^), median (IQR)	25.8 (23.2–29.3) *(m = 37)*
Comorbidities	
ASA status, *n* (%)	
ASA I	45 (16.6%)
ASA II	100 (36.9%)
ASA III	102 (37.6%)
ASA IV	24 (8.9%)
Any comorbidity, n (%)	184 (67.9%)
Diabetes, *n* (%)	66 (24.4%)
Cardiovascular disease, *n* (%)	106 (39.1%)
Chronic kidney disease, *n* (%)	24 (8.9%)
Active malignancy, *n* (%)	36 (13.3%)
Immunodeficiency, *n* (%)	49 (18.1%)
Liver disease, *n* (%)	11 (4.2%) *(m = 9)*
COPD, *n* (%)	32 (12.2%) *(m = 9)*
Connective tissue disease, *n* (%)	16 (6.1%) *(m = 9)*
Site of infection^a^	
Head/neck, *n* (%)	17 (6.5%) *(m = 8)*
Upper extremity/thorax, *n* (%)	52 (19.8%) *(m = 8)*
Abdomen, *n* (%)	31 (11.8%) *(m = 8)*
Anogenital/gluteal, *n* (%)	84 (31.9%) *(m = 8)*
Lower extremity, *n* (%)	79 (30.0%) *(m = 8)*

*BMI *Body Mass Index, *COPD* chronic obstructive pulmonary disease, *NSTI* necrotizing soft tissue infection. ^a^Refers to the first anatomical area affected by the infection. *m* = missing

**Table 2:** Association of comorbidities with clinical outcomes

Comorbidity	In-hospital mortality with	In-hospital mortality without	*p*-value	Amputation with	Amputation without	*p*-value	Mechanical ventilation with	Mechanical ventilation without	*p*-value
Diabetes, *n* (%)	17/66 (25.8%)	40/205 (19.5%)	0.279	9/65 (13.8%)	22/198 (11.1%)	0.553	46/63 (73.0%)	131/183 (71.6%)	0.827
Cardiovascular disease, *n* (%)	36/106 (34.0%)	21/165 (12.7%)	< 0.001	14/103 (13.6%)	17/160 (10.6%)	0.466	74/99 (74.4%)	103/147 (70.1%)	0.423
Chronic kidney disease, *n* (%)	14/24 (58.3%)	43/247 (17.4%)	< 0.001	4/23 (17.4%)	27/240 (11.3%)	0.383	16/23 (69.6%)	161/223 (72.2%)	0.789
Active malignancy, *n* (%)	15/36 (41.7%)	42/235 (17.9%)	0.001	4/32 (12.5%)	27/231 (11.7%)	0.777	23/32 (71.9%)	154/214 (72.0%)	0.992
Immunodeficiency, *n* (%)	17/49 (34.7%)	40/222 (18.0%)	0.010	6/44 (13.6%)	25/219 (11.4%)	0.677	34/44 (77.3%)	143/202 (70.8%)	0.386
Liver disease, *n* (%)	5/11 (45.5%)	51/251 (20.3%)	0.061	1/11 (9.1%)	29/243 (11.9%)	1.00	9/9 (100.0%)	165/232 (71.1%)	0.058
COPD, *n* (%)	6/32 (18.8%)	50/230 (21.7%)	0.699	2/31 (6.5%)	28/223 (12.6%)	0.551	18/28 (64.3%)	156/213 (73.2%)	0.320
Connective tissue disease, *n* (%)	6/16 (37.5%)	50/246 (20.3%)	0.104	3/16 (18.8%)	27/238 (11.3%)	0.414	14/15 (93.3%)	160/226 (70.8%)	0.074

*COPD* chronic obstructive pulmonary disease

### Symptoms at hospital presentation

**Fig. 1:**
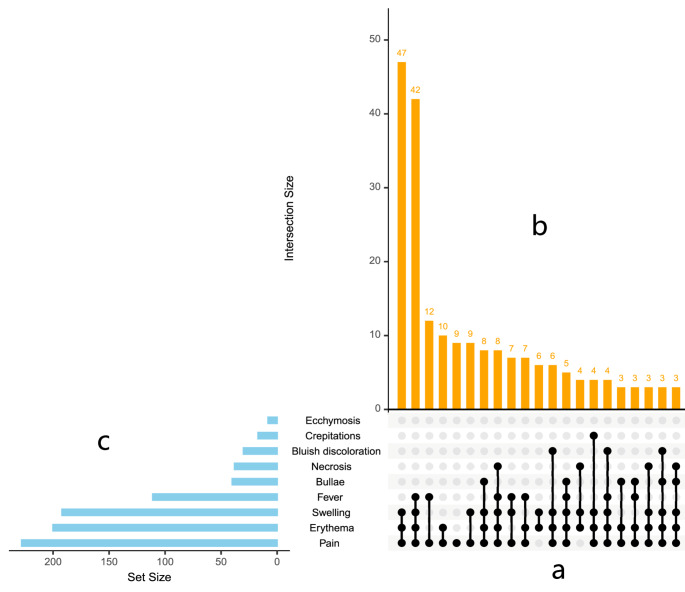
Prevalence of symptom combinations at hospital presentation. Dots connected by lines (**A**) below the bars illustrate which symptoms are present in each combination. The bar height (**B**) indicates the number of cases with each symptom combination. The set size (**C**) displays the number of cases that presented with each symptom. Symptom combinations that were present in fewer than three cases were excluded. There was missing data for 13 cases (4.8%) except for the variable fever, for which there was missing data for 17 cases (6.3%)

Overall, pain was the most frequently reported symptom (88.4%), followed by erythema (77.5%), swelling (74.4%), and fever (43.7%). Bullae (15.5%), skin necrosis (14.7%), blue-grey skin discoloration (11.6%), crepitation upon palpation (6.6%), and ecchymosis (2.7%) were described less frequently. The most frequent symptom combinations were pain, erythema, and swelling (17.3%), followed by this specific combination with the addition of fever (15.5%) (Fig. 1). Pain in combination with fever was observed in 4.4%, pain in combination with erythema in 3.7%, and pain as the only symptom in 3.3%. Septic shock occurred in 56.6% of cases.

At initial hospital presentation, 60.9% of patients presented with early-stage, nonspecific NSTI symptoms (erythema, pain, swelling, or fever), whereas 39.1% of patients also presented with additional late-stage symptoms (bullae, skin necrosis, blue-grey skin discoloration, crepitation upon palpation, or ecchymosis). Patients presenting with early-stage symptoms were significantly more misdiagnosed (60.5%) than those presenting with additional late-stage symptoms (45.4%; p = *.019*). Additionally, patients presenting with early-stage symptoms experienced a significantly longer interval from admission to the first debridement (11 h, IQR 5–29), compared to those also presenting with late-stage symptoms (6 h, IQR 4–13; p = *.009*). A portal of entry was visible in 68.9% of patients. A visible portal of entry was significantly more common in patients with polymicrobial NSTI (75.2%) compared to those with monomicrobial NSTI (60.8%; *p* =.025). Patients with a visible portal of entry presented with early-stage symptoms in 58.3% of cases, whereas 41.7% of cases also had late-stage symptoms (p = *.037*). Among those without a visible portal of entry, 67.1% presented with early-stage symptoms, while 32.9% presented with additional late-stage symptoms (p = *.004*). The misdiagnosis rate was similar for patients with (53.3%) and without (56.0%) a visible portal of entry (*p* =.609).

### Microbiological findings

**Fig. 2:**
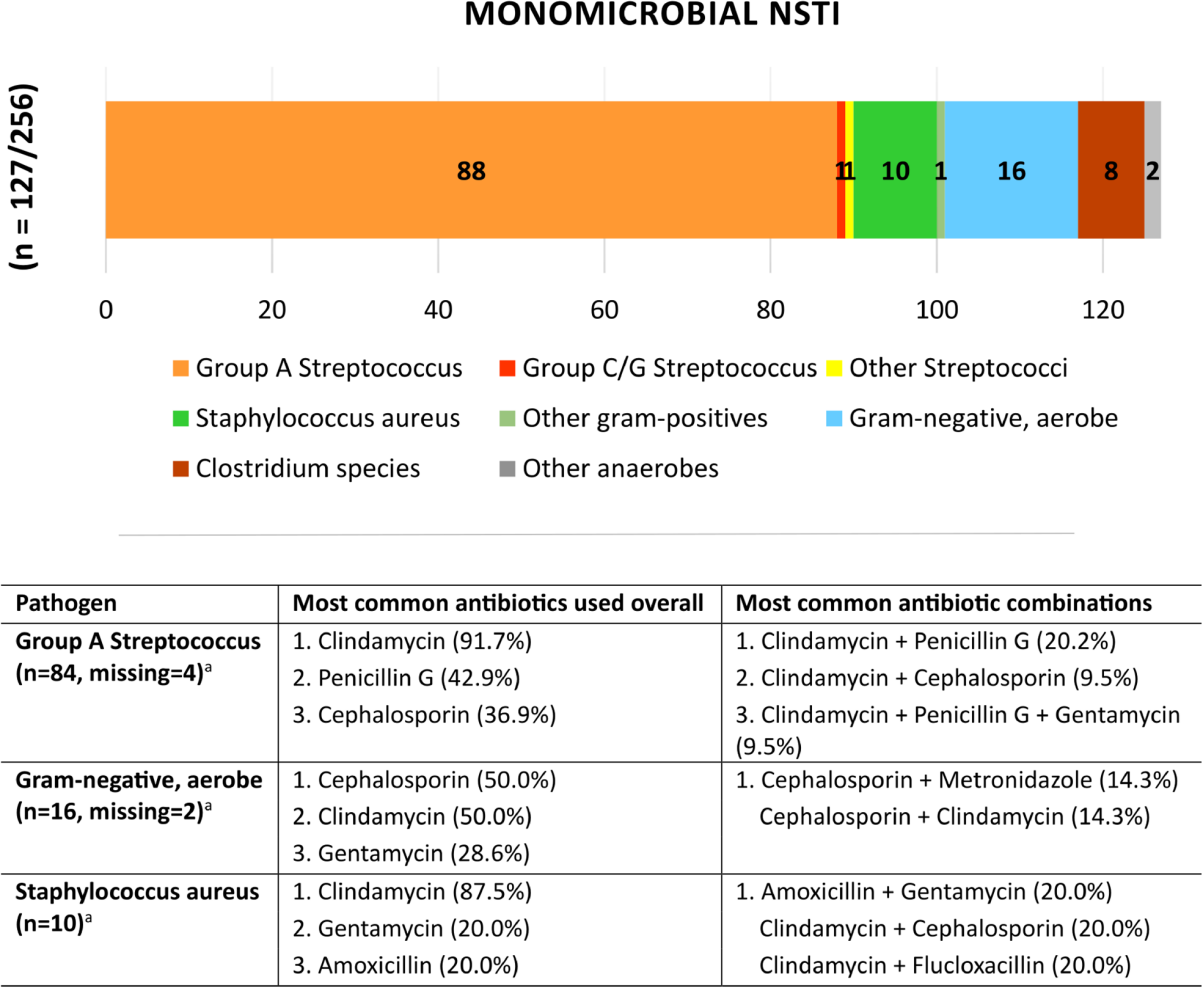
Cultured pathogens and antibiotics used in monomicrobial NSTI. The most commonly administered antibiotic combinations and individual antibiotics following the working diagnosis of monomicrobial NSTI are presented separately for the three most common pathogens. Only the three most common antibiotics and antibiotic combinations are reported. Combinations used in only one patient were excluded. ^**a**^Missing values refer to antibiotic data, not pathogen identification

**Fig. 3:**
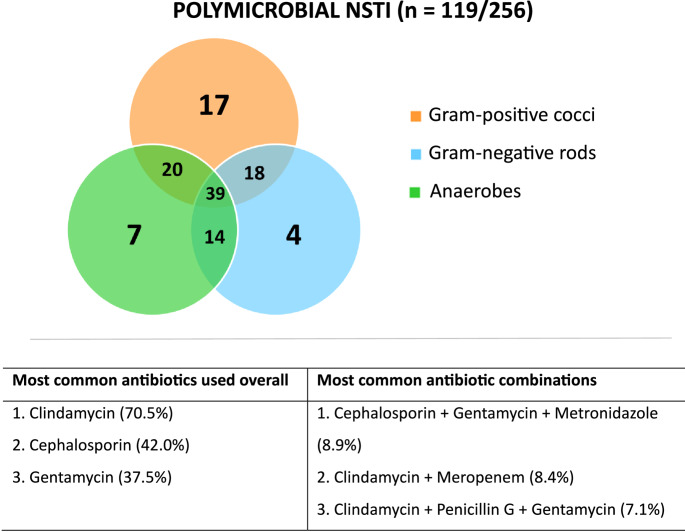
Cultured pathogens and antibiotics used in polymicrobial NSTI. There was missing data regarding antibiotic use for 7 cases (5.9%). Only the three most common antibiotics and antibiotic combinations were reported. Clindamycin was the most frequently used antibiotic overall, but was administered in a wide variety of combinations

Bacteria culture test results showed that NSTI was monomicrobial in 49.6% and polymicrobial in 46.5% of cases (p = *.655*). GAS accounted for 69.3% of the monomicrobial cases and 41.4% of cases overall. In 3.9% of cases, no pathogen could be cultured. A specification of cultured pathogens is presented separately for monomicrobial and polymicrobial NSTI in Figs. 2 and 3, respectively.

### Laboratory results and treatment characteristics

The laboratory results at hospital presentation are presented in Table 3. The median LRINEC score at hospital presentation was 8 (IQR 5–9). The most commonly administered antibiotics and antibiotic combinations following the working diagnosis of NSTI are presented separately for monomicrobial and polymicrobial cases (Figs. 2 and 3). Three patients (1.1%) received hyperbaric oxygen therapy (HBOT), whereas intravenous immunoglobulins (IVIG) were administered to 45 patients (16.7%) (Table 3). The median time from hospital admission to the initiation of IVIG treatment was 14 h (IQR 6–26), with a median treatment duration of 3 days (IQR 2–3). Among the 45 patients who received IVIG treatment, 72.7% had GAS cultured. Of all patients with both sepsis and confirmed GAS infection, 48.3% received IVIG. In-hospital mortality among these patients was 21.4%, compared to 20.0% in patients with sepsis and GAS who did not receive IVIG (p = *.964*).

Among all patients diagnosed with NSTI, 82.9% required ICU admission, with a median ICU duration of 5 days (IQR 2–11). Among the patients who survived ICU admission (*n* = 182), the median duration of ICU admission was 6 days (IQR 3–12). Of the patients admitted to the ICU, 86.3% required mechanical ventilation, 85.0% received vasopressor support, and 18.0% underwent CVVH. The median time from admission to the first debridement was 8 h (IQR 4–23). Patients underwent a median of three debridement surgeries (IQR 2–4), with a median of 2% (IQR 1–7) of the Total Body Surface Area (TBSA) resected. Skin-sparing debridement was described in 87 patients (32.1%). The median length of stay (LOS) across all hospital admissions was 23 days (IQR 10–41).

### Clinical outcomes

The in-hospital mortality rate was 21.0%. Mortality rates were 19.5% at 30 days and 25.5% at 90 days. The in-hospital mortality rate did not differ significantly between patients presenting with early-stage (22.3%) and additional late-stage NSTI symptoms (21.8%; p = *.923*). However, among the patients who only presented with early-stage symptoms, those who were misdiagnosed had a significantly higher in-hospital mortality rate (30.4%) than patients who were correctly diagnosed (10.0%; p = *.003*). No significant difference in in-hospital mortality was observed between patients with monomicrobial (17.3%) and polymicrobial NSTI (21.8%; p = *.371*).

A total of 36 (partial) extremity amputations were performed in 31 patients (11.8%), which are further specified in Table 3. Of all extremity amputations, 14 procedures (38.9%) were performed during the first debridement, 9 (25.0%) during the second debridement, and 5 (13.9%) during the third debridement. The remaining 8 amputations (22.2%) were performed during subsequent debridement procedures. There was no significant difference in the amputation rate between patients presenting with early-stage (8.4%) and late-stage NSTI symptoms (16.7%; p = *.089*), as well as between patients who were misdiagnosed (10.2%) and correctly diagnosed (14.6%; p = *.310*). However, septic shock occurred significantly more often in patients who underwent extremity amputation (80.0%) compared to those who did not (53.9%; p = *.005*). Among patients with monomicrobial NSTI, 15.9% underwent amputation, compared to 8.8% of patients with polymicrobial NSTI (*p* =.052).

**Table 3:** Laboratory results, treatment characteristics, and clinical outcomes

	*N* = 271
Laboratory results at presentation	
CRP (mg/L), median (IQR)	297 (190–395) *(m = 40)*
Leukocytes (x10^9^), median (IQR)	16 (9–22) *(m = 39)*
Hemoglobin (mmol/L), median (IQR)	8 (7–9) *(m = 45)*
Creatinine (µmol/L), median (IQR)	116 (79–197) *(m = 43)*
Sodium (mmol/L), median (IQR)	134 (130–137) *(m = 51)*
Glucose (mmol/L), median (IQR)	8 (6–10) *(m = 73)*
LRINEC score, median (IQR)	8 (5–9) *(m = 75)*
Low risk (score ≤ 5), *n* (%)	57 (29.1%)
Intermediate risk (score 6–7), *n* (%)	39 (19.9%)
High risk (score ≥ 8), *n* (%)	100 (51.0%)
Medical management	
HBOT, *n* (%)	3 (1.1%) *(m = 1)*
IVIG, *n* (%)	45 (16.7%) *(m = 1)*
Interval between admission and start IVIG (hours), median (IQR)	14 (6–26) *(m = 10)*
Days IVIG, median (IQR)	3 (2–3) *(m = 8)*
ICU admission, *n* (%)	223 (82.9%) *(m = 2)*
Mechanical ventilation, *n* (%)	177 (86.3%) *(m = 18)*
Vasopressor support, n (%)	159 (85.0%) *(m = 36)*
CVVH, n (%)	37 (18.0%) *(m = 18)*
ICU duration (days), median (IQR)	5 (2–11) *(m = 7)*
Total LOS (days), median (IQR)	23 (10–41)
Surgical management	
Time from admission to first debridement (hours), median (IQR)	8 (4–23) *(m = 83)*
Number of debridement surgeries, median (IQR)	3 (2–4) *(m = 20)*
TBSA% cutaneous resected, median (IQR)	2 (1–7) *(m = 82)*
Skin-sparing debridement, *n* (%)	87 (32.1%)
Negative pressure therapy, *n* (%)	131 (51.8%) *(m = 18)*
Clinical outcomes	
Extremity amputation, *n* (%)	31 (11.8%) *(m = 8)*
Amputation procedures, *n* (%)	36 (100%)
Upper arm, *n* (%)	3 (8.3%)
Fingers, *n* (%)	5 (13.9%)
Upper leg, *n* (%)	13 (36.1%)
Lower leg, *n* (%)	11 (30.6%)
Foot, *n* (%)	1 (2.8%)
Hallux, *n* (%)	1 (2.8%)
Toes, *n* (%)	2 (5.6%)
In-hospital mortality, *n* (%)	57 (21.0%)
30-day mortality, *n* (%)	51 (19.5%) *(m = 9)*
90-day mortality, *n* (%)	64 (25.5%) *(m = 20)*

*CVVH* Continuous Veno-Venous Hemofiltration, *HBOT* hyperbaric oxygen therapy, *ICU *intensive care unit, *IVIG* intravenous immunoglobulins,* LOS *length of stay, *TBSA* total body surface area. Amputation of a body part may be either partial or complete. *m* = missing

## Discussion

This study demonstrated that in the Netherlands, both mortality and morbidity of patients with NSTI are substantial. Over one in five patients died, and one in eight patients required (partial) amputation of a limb. The findings of our study also highlighted the significant diagnostic challenges posed by NSTI, particularly in its early stages, when patients often present with nonspecific symptoms. Patients who presented with these symptoms were misdiagnosed more frequently and experienced a longer interval to the first debridement than patients who presented with late-stage symptoms. Moreover, patients presenting with early-stage symptoms who were misdiagnosed had a significantly higher in-hospital mortality rate compared to those who were accurately diagnosed, stressing the need for earlier recognition of NSTI by healthcare providers.

The mortality rate observed in this study aligns closely with that of another Dutch study (2014–2019), which found rates ranging from 23% to 29% [11]. It is also consistent with retrospective studies conducted in Denmark and France, but higher than reported in the INFECT trial (14% at 30 days and 18% at 90 days) [1, 6, 10]. A possible explanation for this discrepancy is that in our cohort, septic shock was considerably more common (82.9%) than in the INFECT trial (50.0%). In addition, in the INFECT trial, a substantially larger proportion of patients received HBOT (80%) and IVIG treatment (58%), compared to 1.1% and 16.7% in this cohort, respectively. However, robust scientific evidence for these treatments remains limited, and in the current cohort, the mortality rate of septic patients who received IVIG treatment was comparable to those who did not [17, 18]. Nevertheless, given that only 48.3% of patients with GAS and sepsis received IVIG, and only 1.1% of patients overall received HBOT, it is possible that broader use of these treatments could have resulted in improved outcomes. However, to confirm this assumption, randomized controlled trials are needed to evaluate the effectiveness of IVIG specifically in GAS-related cases, as well as assess the role of HBOT in the broader NSTI population.

The rate of extremity amputation in this study was 11.8%, which is consistent with rates previously reported in Dutch studies (10%−14%) [11, 12]. Internationally, a wider amputation rate is reported, ranging from 6% to 34% [19, 20]. Notably, in our cohort, 38.8% of amputations were performed during the initial debridement procedure, with nearly all (80.0%) of the patients experiencing septic shock. These findings suggest that early amputation is likely driven by the urgent need for source control rather than irreparable tissue damage, in which case a delayed amputation procedure would be expected. Effective source control with maximal preservation of viable tissue is most effectively achieved by the skin-sparing debridement technique [21]. Indocyanine green angiography, which enables real-time assessment of tissue viability, may prevent unnecessary amputations even further by increasing debridement precision and minimizing tissue loss [22].

Notably, in our cohort, most patients presented with non-specific early-stage NSTI symptoms, typically pain, erythema, and swelling. These symptoms closely resemble non-necrotizing soft tissue infections, making their distinction challenging. A portal of entry was less frequently encountered in monomicrobial infections compared to polymicrobial infections, which is in line with previous findings describing that up to half of the NSTI cases due to GAS, the most frequent causative pathogen in monomicrobial NSTI, may be caused by hematogenous spread from the nose, throat, or ear [15]. Additionally, patients with a visible portal of entry were found to more frequently present with late-stage symptoms compared to patients without a portal of entry. This may partly be explained by the earlier onset of visible skin manifestations in patients with a portal of entry, so that by the time of presentation, these symptoms have already progressed to late-stage features [15]. Although the in-hospital mortality did not differ significantly between patients presenting with early-stage symptoms and patients with additional late-stage symptoms, the in-hospital mortality of patients with early-stage symptoms who were misdiagnosed was substantially higher. This suggests that timely recognition and correct diagnosis influence patient outcomes more than the stage of presentation alone. While computed tomography and the LRINEC score can support diagnosis, their diagnostic performance is limited [23, 24]. In the current study, 29% of patients were incorrectly classified by the LRINEC score as low risk. Magnetic resonance imaging is highly sensitive, although its application is often not feasible in an acute care setting. Emerging diagnostic techniques such as point-of-care ultrasound and artificial intelligence-based screening tools may prove useful in distinguishing NSTI from less severe infections [25, 26]. Because NSTI is primarily a clinical diagnosis, improving recognition requires strategies to improve clinician awareness of the disease itself and early symptom clusters. Potential strategies to improve this awareness could be providing education and establishing NSTI expertise networks. Additionally, implementing structured soft tissue infection protocols in emergency departments could help reduce diagnostic delays and improve outcomes. If healthcare providers recognize the disease early, surgical exploration can be performed, followed by debridement when the diagnosis is confirmed.

Notably, in our study, GAS was cultured in 41.4% of patients, which is higher than the European average of 24.9% reported in a recent systematic review (2020), but consistent with a previous Dutch study [11, 27]. Since March 2022, the Dutch National Institute for Public Health and the Environment (RIVM) has reported an increase in invasive GAS (iGAS) related infections in the Netherlands. This trend was initially observed among children aged 0 to 5 years, but later extended to adults, contributing to a rise in the incidence of necrotizing fasciitis [28]. This trend has also been observed in several other European countries, including the United Kingdom, France, Ireland, Denmark, and Sweden [29, 30]. The cause remains unclear, although some researchers suggest it may be linked to an “immunity debt” created by reduced exposure to pathogens during the COVID-19 pandemic [31]. It would be interesting for future nationwide studies to focus on the incidence of NSTI caused by GAS and compare these findings with our results, along with a detailed analysis of characteristics and clinical outcomes.

This study has several strengths, including its multicenter design and the extensive range of variables collected, which give a thorough overview of the cohort. Additionally, the comprehensive search strategy used to identify patients in the EHR likely contributed to a complete capture of all NSTI diagnoses. However, limitations should be considered. Although we assume this is a representative cohort, it remains a sample of the total population, with a relative overrepresentation of academic hospitals and burn centers compared to peripheral hospitals. Therefore, our findings may not fully reflect the true national characteristics. Second, as this study was retrospective and based on data from the EHR, despite our best efforts, the proportion of missing data for certain parameters was substantial, which possibly influenced our results. For instance, the 30% missing data on the percentage of TBSA resected could result in an overestimation of this variable, as larger TBSAs might be documented more often compared to smaller, less clinically significant ones. Additionally, it is unclear how much missing data there is for the symptoms of presentation, as the absence of a symptom in the EHR does not necessarily indicate that it was not present.

With the apparent increase of GAS-related NSTI infections in recent years, it would be insightful for future studies to examine whether other characteristics and outcomes have also shifted, and whether this increase normalizes again. This current historical cohort can serve as a reference for such studies. Furthermore, establishing a nationwide prospective registration platform with a core outcome set would enable standardized and comprehensive data collection, allowing the identification of trends over time and serving as a foundation for future research. This also enables us to evaluate whether our efforts to improve NSTI care have translated into better patient outcomes. To achieve these goals, our research group has established a national NSTI network across multiple Dutch hospitals and is currently recruiting additional centers to achieve nationwide coverage in the future.

## Conclusions

Between 2013 and 2017, one in five NSTI patients who were treated in participating Dutch study centers died, and one in eight patients required amputation of an extremity. NSTI patients demonstrated considerable variation in comorbidities, symptoms of presentation, and microbiological profiles. The findings of this study highlight the substantial potential for improvement of outcomes for NSTI patients and the importance of developments leading to timely diagnosis and treatment. This historical cohort can serve as a reference for future data, helping to evaluate whether interventions have contributed to improved outcomes for NSTI patients.

## Data Availability

Data are available from the corresponding author upon reasonable request.
